# Complete Genome Sequences of Mycobacteriophages TribleTrouble, TClif, Llorens and CallaLilly

**DOI:** 10.17912/micropub.biology.002251

**Published:** 2026-08-11

**Authors:** Sofia Sileo, Daniela Lopez Llorens, Alaina Bowers, Trisha Chhabra, Natalie Clark, Himani Gangumolu, Lucy Gast, Madison Gicale, Vidhi Grover, Roby Hardesty, Kaylee Hutchison, Sanam Krishnani, Landon Patton, Logan Robinson, Ash Sheehan, Shelby Spencer, Nicholas Sugimoto, Simon Sy, Sophia Thomas, Claire A. Rinehart, Rodney A. King

**Affiliations:** 1 Biological Sciences, Western Kentucky University, Bowling Green, KY, United States

## Abstract

We report the genome sequences of four novel phages that infect *Mycobacterium smegmatis* mc ^2^ 155. Phages TribleTrouble, TClif, Llorens and CallaLilly have siphovirus morphology and double-stranded DNA genomes consisting of 61,665bp, 61,466bp, 59,708bp and 59,631bp, respectively. Based on gene content similarity, these phages belong to the K cluster of Mycobacterium phages. No novel genomic features were noted beyond those previously observed for phages of the K cluster.

**Figure 1. Transmission electron microscopy images of phages:** Negative stain (1% uranyl acetate) transmission electron microscopy images show a siphovirus morphology with an icosahedral capsid and a flexible tail. See Table 1 for particle dimensions. Top row: TribleTrouble (left) and TClif (right). Bottom row: Llorens (left) and CallaLilly (right). Table: Phage characteristics, sequencing data and annotation results

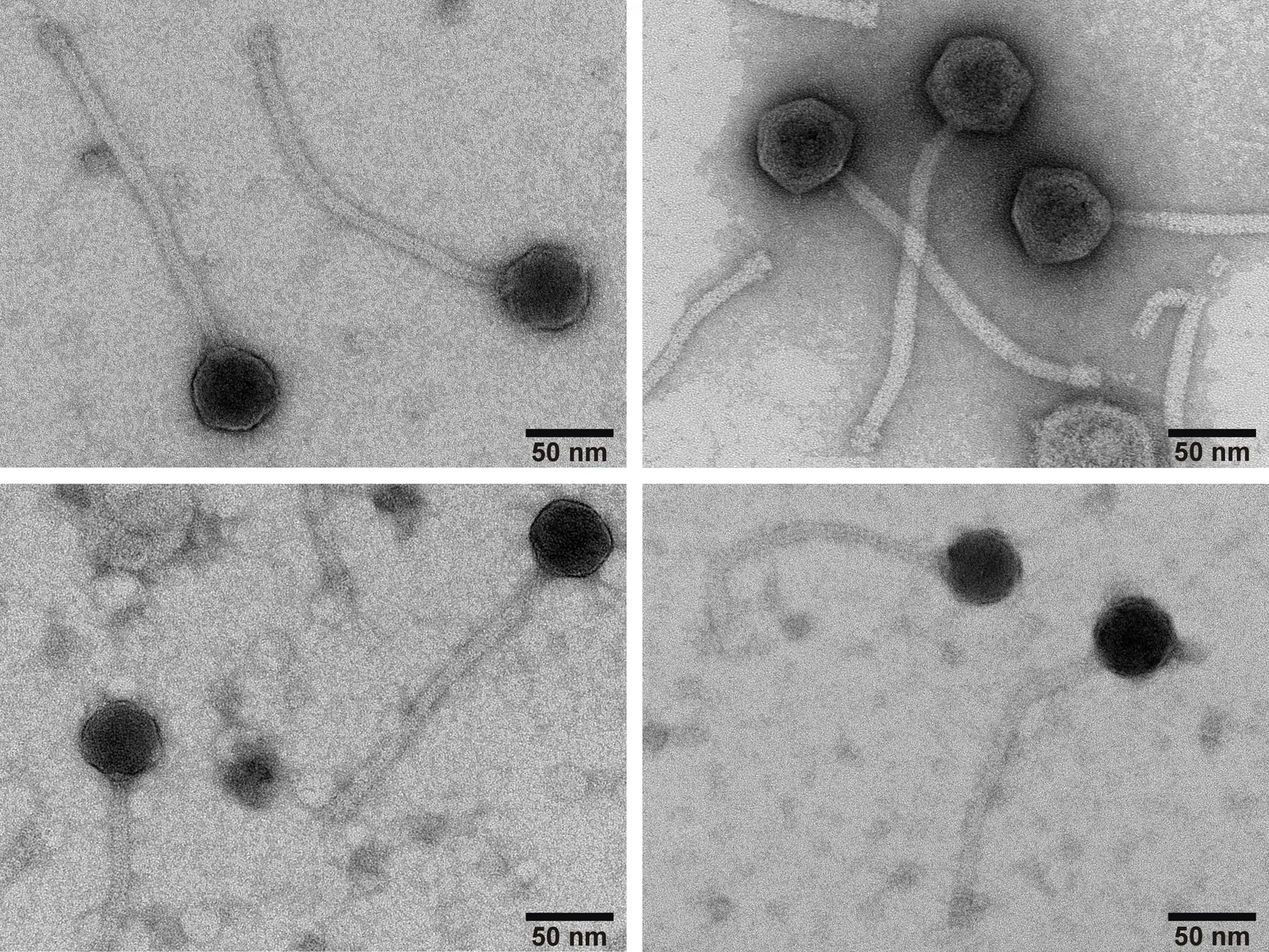



**Table:** 

Phage	TribleTrouble	TClif	Llorens	CallaLilly
Sample Location (City, State)	Bowling Green, KY	Bowling Green, KY	Oak Grove, KY	Bowling Green, KY
Sample location GPS coordinates	36.986335 N, 86.454495 W	37.000674 N, 86.428339 W	36.64956 N, 87.43688 W	36.986367 N, 86.454448 W
Predicted lifestyle	Temperate	Temperate	Temperate	Temperate
Plaque Morphology	Turbid	Turbid	Turbid	Turbid
Plaque Size (mm)	1-2 (n = 25)	2-5 (n=5)	1-3 (n= 12)	3-4 (n=10)
Capsid Size (nm)	55.5 +/- 3.5, n=29	59.7 +/- 2.7, n=10	57.6 +/- 5.7, n=42	60.7 +/- 5.3, n=10
Tail Length (nm)	234 +/- 14.2, n=29	200.1 +/- 4.8, n=10	211.1 +/- 20.6, n=42	204.1 +/- 9.7, n=10
SRA accession number	SRX31241832	SRX31241831	SRX31241839	SRX31241827
# of reads	737,718	813,727	813,737	1,833,034
Approximate read coverage	1,913	1,865	1,925	3,024
Genbank accession number	OR475294	PQ201077	PQ559665	PV915854
Cluster	K3	K6	K1	K1
Genome length (bp)	61,665	61,466	59,708	59,631
GC content (%)	66.4	67.5	66.5	66.5
Genome termini	11 base, 3' single stranded overhang	11 base, 3' single stranded overhang	11 base, 3' single stranded overhang	11 base, 3' single stranded overhang
Number of tRNA genes	0	0	1	1
Number of ORFs	97	100	95	95

## Description

Mycobacteriophages represent a rich source of viral genomic diversity, and their sequencing supports both fundamental biological insights and educational programs like the Science Education Alliance Phage Hunters Advancing Genomics and Evolutionary Science (SEA-PHAGES) initiative (Jordan et al., 2014). A deeper understanding of the genetic diversity of bacteriophage genomes may reveal novel genes whose products could have applications in medicine and biotechnology. The genomes of four double-stranded DNA (dsDNA)-tailed bacteriophages (TribleTrouble, TClif, Llorens, and CallaLilly) that infect *Mycobacterium smegmatis* mc ^2^ 155 are reported here. These phages provide additional representation of cluster K phages and facilitate comparative analyses of genome architecture, gene content, and structural features.

Phages were isolated using standard enrichment techniques (Zorawik et al., 2024). Briefly, soil samples were resuspended in Middlebrook 7H9 liquid medium, inoculated with *M. smegmatis* mc ^2^ 155 and incubated with shaking at 30˚C. After 48 hours, the cultures were centrifuged, the supernatant was filtered (0.2 μm pore filter) and the filtrate was plated in top agar with *M. smegmatis.* Plates were incubated at 30˚C for 48 hours. All phages were purified through a minimum of three rounds of plaque assay.

Electron microscopy on negatively stained (uranyl acetate, 1%) particles revealed the phages have siphovirus morphology (Figure 1). Phage genomic DNA was extracted from a lysate using the Promega Wizard Cleanup Kit. Libraries were prepared using the NEB Ultra II FS kit, and sequenced on an Ilumina MiSeq 1000 (TribleTrouble, TClif and Llorens; v3 reagents) or a NextSeq1000 (CallaLilly; XLEAP-P1 kit). The raw reads generated on the MiSeq1000 were assembled with Newbler v2.9 to generate single contigs. The raw reads generated on the NextSeq1000 were trimmed with cutadapt 4.7 (using the option: –nextseq-trim 30) and filtered with skewer 0.2.2 (using the options: -q 20 -Q 30 -n -l 50) prior to assembly (Martin, 2011, Jiang et al., 2014, Wick et al, 2017). The genomes were checked for completeness and termini type using Consed v29 (Gordon and Green, 2013, Russell, 2017). Additional sequencing and genome details are summarized in Table 1.

Genes were predicted using Glimmer v3.02 (Delcher et al., 2007), Genemark.hmm v3.36, GenemarkS v4.28 (Besemer and Borodovsky, 2005), Aragorn v1.2.38 (Laslett and Canback, 2004) and tRNAscan-SE v2.0.12 (Lowe and Eddy, 1997). Manual inspection and revision were performed using PECAAN v20250130 (Rinehart et al, 2016). Within PECAAN, gene functions were assigned using HHPRED (Söding et al., 2005) alignment to the PDB_mmCIF70, Pfam- v.36, NCBI’s Conserved Domains databases and BLAST (Altschul et al., 1990), alignment to the NCBI nonredundant protein (National Center for Biotechnology Information) and PhagesDB (Russell and Hatfull, 2016) databases. Default settings were used for all software. Based on gene content similarity the phages were assigned to specific actinobacteriophage subclusters (Table 1) (Pope et al, 2017; Russell and Hatfull, 2016).

Functions were predicted for approximately 49% of the called genes across all four phages. Structural proteins (e.g. major capsid and tail proteins) are located in the left region of the genomes, followed by the lysis cassette, which includes the lysin A, holin, and lysin B genes. The predicted immunity repressor of all four phages belong to the same pham; a feature shared with phages known to form lysogens. (Cresawn et. al., 2011, Pope et al., 2011). Similarly, the predicted tyrosine integrases of TClif, Llorens and CallaLilly and the cluster K phages known to form lysogens all belong to the same pham. TribleTrouble encodes a predicted tyrosine integrase that belongs to a different pham, but is present in phages previously shown to establish lysogeny (e.g. Fionnbharth). Predicted DNA metabolism and replication genes are located on the right arm of the genome and nearly all of the predicted genes are transcribed in the forward direction. The exceptions include the predicted immunity repressor, a membrane protein, a secreted protein, and a tRNA gene located in the Llorens and CallaLilly genomes. Although phages tend to display high host specificity, members of the K1 subcluster have been shown to infect both fast- and slow-growing mycobacterial hosts (Pope et al., 2011).

**Data availability:**

GenBank and Sequence Read Archive (SRA) accession numbers are provided in Table 1
